# Editorial: Computational Systems Biology of Pathogen-Host Interactions

**DOI:** 10.3389/fmicb.2016.00021

**Published:** 2016-02-04

**Authors:** Saliha Durmuş, Tunahan Çakır, Reinhard Guthke

**Affiliations:** ^1^Computational Systems Biology Group, Department of Bioengineering, Gebze Technical UniversityKocaeli, Turkey; ^2^Leibniz Institute for Natural Product Research and Infection Biology - Hans Knöll InstituteJena, Germany

**Keywords:** pathogen-host interaction, computational systems biology, bioinformatics, omics data, network inference, text mining, constraint-based modeling, image-based systems biology

Pathogen-Host Interactions (PHIs) play a significant role in the mechanisms of infections. Therefore, the investigation of infection mechanisms through PHIs is a crucial step to develop novel and more effective solutions against drug-resistance and for personalized therapy. To this aim, systems biology approach considers the whole PHI system instead of focusing hosts or pathogens individually. Computational modeling and analysis has a vital place within the whole systems biology workflow (Cyclic operation of experimental and modeling work). Multi-scale modeling provides the holistic view needed in the investigation of pathogen-host molecular interactions. However, it is usually very difficult to identify the model structure and parameters for complex multi-scale models. On the other hand, focused modeling types require more stringent and advanced feature selection approaches.

This research topic aims to provide examples from the current picture of the research on computational systems biology of PHIs. The papers included here review recent studies or present original research on computational inference of PHI networks, computational prediction of PHIs, text mining of PHI data from the literature, and mathematical modeling and computational analysis of PHI networks. This research topic presents three review papers, 10 original research articles, and one technology report.

Opening this research topic, we provide a comprehensive review of the studies on computational systems biology of PHIs (Durmuş et al.). We focus on the computational methods for the inference of molecular interaction networks of PHI systems, bioinformatic analysis of PHI networks, the Web-based PHI databases, and text-mining efforts to extract PHI data hidden in the literature. In this sense, this review provides a systems perspective on which the other articles covered in this research topic are based.

## PHI network inference using omics data

Schulze et al. deal with the challenge of the inference of inter-species gene regulatory networks from dual transcriptomic data. They use an extended version of NetGenerator, an ordinary differential equations (ODEs)-based tool for network inference that predicts gene regulatory networks from gene expression time series data (Guthke et al., 2005; Tierney et al., 2012).

Budak et al. use a temporal phosphoproteomic dataset of Salmonella-infected human cells (Rogers et al., 2011) to reconstruct the temporal signaling network of the human host by integrating protein-protein interaction (PPI) and the phosphoproteomic data. The Prize-collecting Steiner Forest approach and the Integer Linear Programming based edge inference approach are employed. The complementary use of both methods leads to a network which conserves the information about temporality, direction of interactions, while revealing the hidden entities in the signaling.

## Computational prediction of PHIs

Despite the recent advances, the experimentally-found PHI data are still scarce and the computational prediction is a valuable source of PHI data currently. The computational prediction primarily exploits sequence information, protein structure and known interactions. Machine learning techniques are used when there are sufficient known interactions available to be used as training data. On the opposite case, transfer and multitask learning methods are preferred. Nourani et al. provide an overview of these approaches for predicting PHIs.

Experimentally verified data on fungi-host interactions are rare in the literature and in the PHI databases. Remmele et al. reconstruct large-scale PHI networks for the fungal pathogens *Aspergillus fumigatus* and *Candida albicans* and their human and mouse hosts. A computational PHI prediction method based on protein orthology, PPI data as well as data on gene functions and cellular localization was developed and used.

## Text mining of PHI data

The emergence of large-scale experimental PHI data has led to the development of PHI databases such as VirusMentha (Calderone et al., 2015), VirhostNet (Guirimand et al., 2015), PATRIC (Wattam et al., 2014), HPIDB (Kumar and Nanduri, 2010), and PHISTO (Durmuş Tekir et al., 2013). Nevertheless, most data regarding PHIs are still buried in the articles and they have not been stored in databases. Karadeniz et al. extend text mining tool SciMiner, originally developed for extracting intra-species molecular interactions, for inter-species PHIs. They use SciMiner to extract host-*Brucella* gene-gene interactions, which are further analyzed by ontology modeling.

## Mathematical modeling and bioinformatic analysis of PHIs

Few examples of constraint-based PHI models are currently available in the literature. However, there is a lack of definite description of the methodology required for the functional integration of genome scale metabolic models in order to generate PHI models. Jamshidi and Raghunathan outline a systematic procedure to produce functional PHI models, highlighting steps which require debugging and iterative revisions in order to successfully build a functional model. The construction of such models will enable the exploration of PHIs by leveraging the growing wealth of omics data in order to better understand mechanisms of infection and identify novel therapeutic strategies.

Dühring et al. describe the cross-talk between the fungal pathogen *C. albicans* and the human innate immune system. They review computational systems biology approaches to model and investigate these complex interactions with a special focus on fungal immune evasion and game-theoretical and agent-based models.

Nguyen et al. use ODEs to represent the basic interactions between Ebola virus and wild-type Vero cells, i.e., epithelial cells of green monkeys, *in vitro*. The parameters in viral kinetics are estimated leading to a first mathematical model for Ebola virus infection.

Dix et al. examine the transcriptional footprint of the host in response to the bacterial pathogens *Staphylococcus aureus* and *Escherichia coli* and the fungal pathogens *C. albicans* and *A. fumigatus* in a human whole-blood model. Expression data are exploited to build a random forest classifier to classify if a sample contains a bacterial, fungal or mock-infection.

Sinclair et al. develop a method combining *in silico* prediction of bacterial nucleomodulins, i.e., proteins targeted to the host cell nucleus, and iTRAQ protein profiling (a mass spectrometric technique where two protein expression profiles are compared) to identify potential bacterial-derived nuclear-translocated proteins that could impact transcriptional programming in host cells. This approach was applied to intracellular bacteria such as *Anaplasma phagocytophilum, Mycobacterium tuberculosis*, and *Chlamydia trachomatis.*

Finally, the research topic includes articles focusing on image-based systems biology of PHIs. While advances in omics techniques drive the progress of system biology on molecular level, there is also a significant progress on the cellular level based spatio-temporal data, e.g., microscopy images. Lehnert et al. apply non-spatial state-based modeling and agent-based modeling approaches to simulate an experimental assay for *C. albicans* infection of human blood. They predict cell migration parameters in 3D space where monocytes, granulocytes, and *C. albicans* cells are treated as migrating and interacting agents. Pollmächer and Figge implement a hybrid agent-based spatio-temporal modeling approach for *A. fumigatus* infection in human alveoli to decipher chemokine properties. They found by model simulations that the ratio of chemokine secretion rate to the diffusion coefficient is the main indicator for the success of pathogen detection by alveolar macrophages. Kraibooj et al. suggest a novel image analysis algorithm for the automated quantification of the phagocytosis of two wild type *A. fumigatus* strains. The strains were compared in terms of the phagocytosis process when the fungal conidia interact with alveolar macrophages.

The computational modeling of PHI networks of interacting genes, transcripts, proteins, and metabolites is crucial to enlighten the molecular mechanisms of infection. The experimental detection of levels of biomolecules via omics approaches as well as the detection of PHIs via high-throughput experiments started to generate comprehensive datasets. The modeling of the large-scale data will not only elucidate the mechanisms of infection, but will help in the discovery of biomarkers for novel diagnostic tools and of therapeutic drug targets through identification of essential molecules for the pathogen. Despite the recent efforts, the use of systems biology approaches to investigate PHI systems is still in its infancy, mostly because of data scarcity (Durmuş et al.). Ongoing studies in the field will certainly produce more large-scale PHI data in the near future. Heterogeneous data sets (clinical, microbiological, chemical, molecular on different levels such as SNPs, transcriptome, proteome, FACS, microscopic, mass spectrometric, etc.) will be integrated. More complete PHI models will allow the integration of omics-based and image-based systems biology of infection and will pioneer more complex multi-scale models with different scale in space (from molecules/cells/tissues to organism/population) and time (from seconds to month). These more complex models will improve the PHI-based solutions to infectious diseases.

## Author contributions

SD conceived the content and drafted the manuscript; TC and RG conceived the content and revised the manuscript.

### Conflict of interest statement

The authors declare that the research was conducted in the absence of any commercial or financial relationships that could be construed as a potential conflict of interest.
